# Dr. Thomas G. Morton

**Published:** 1903-06

**Authors:** 


Dr. Thomas G. Morton, of Philadelphia, died at Atlantic City, May 20, 1903, aged 67 years. His death was sudden, after but two days' illness, and was reported due to cholera morbus. Dr. Morton was one of the prominent surgeons of Philadelphia and his loss to the profession and the city is distinctly a great one.

